# Economics of Philanthropy

**Published:** 1898-04-23

**Authors:** 


### The Institutional Workshop.

### ECONOMICS OF PHILANTHROPY.

### VI.—HOSPITALS.

While commending all schemes by which people can be helped to provide for themselves in the emergencies of life, we are forced to admit that cases arise for which it has been practically impossible for those who experience them to be prepared. Life will not conform to our rules, and therefore there must be some provision for contingencies. For the unforeseen contingencies of sickness we have hospitals. Of late years hospitals have been subjected to such a fire of criticism that it is worth while to ask what reason there exists for it, and how, conversely, hospitals can justify their existence.

So far as indoor patients are concerned there is little fault to find. In large towns, and even in smaller ones where any manufacturing industry is carried on, there can be no denying of the need for some place where accidents can be taken and treated with the skill and care which, as a rule, are unattainable in their own homes. To justify this provision it is only necessary to visit the home of the ordinary workman and compare it with a hospital ward. In many cases it may depend on whether a patient is treated at home or in hospital if the outcome of his illness is to be fatal or not. To the extent to which hospitals save the lives of workers for whom no adequate provision could be made at their own homes their position is unassailable. The difference between a private house, even of a kind in which the patient has much better chances than are obtainable by the average working man, and a hospital, is so obvious that we find persons in all ranks who are about to undergo an operation preferring to be treated in a pay hospital. The hospital merely gives to the poorer man the care which his case demands, and which he could under no conceivable circumstances pay for himself. Severe medical cases come under the same rules. Treated at home the patient would probably die, treated in the hospital he has a very good chance of life; it is therefore an obvious duty to give him that chance. And it must be remembered that while the hospital patient pays nothing in money, he justifies his existence by the opportunity he gives medical students of becoming familiar with the different forms of disease. Those who contribute to hospitals are not merely helping the poor; in fact, they are not so much helping the poor as guaranteeing the competency of the doctors who may attend them or their children. It is to be regretted that sensational stories of hospital "experiments" are so often retailed, sometimes in print, but more frequently and more mischievously by the gossip of well-meaning but ignorant persons—ignorant of medical subjects even when not otherwise illiterate. That new modes of treatment, new operations, are tried first in hospitals is not to be denied, is not a thing one wants to deny. Where could they be tried with such good chance of success? Where with such assured care, such good nursing, such scrupulous cleanliness, such strict attention to doctor's orders? New methods must be tried somewhere, and the first attempts are best made under such circumstances as are likely to give the best results. If any patient goes into hospital without knowing that his case will be studied with a scientific eye, let him be at once informed of the fact. If he prefers not to enter it with this knowledge, let him remain at home. In ninety-nine cases out of every hundred he will say, "Try anything you like that gives me a chance of life." The educational value of hospitals is one of their strongest economic claims, and should rather be insisted on than evaded.

But when we deal with the out-patient department the case is not so clear. Even in the in-patient department of many hospitals too little care is exercised in inquiring if the patient is able to pay for treatment, and there are people in good circumstances who are mean enough to take advantage of this; but it is when we come to the out-patients that we find this injustice abounding. It is admitted by all that the vast majority of those who attend the out-patient department of a hospital should go either to the poor-law infirmary or to a private practitioner. So flagrant has this abuse of charity become that some hospitals keep an officer, whose business it is to inquire into the circumstances of all who have come to seek treatment in the out-patient room. It would take many such officers to make the requisite inquiries in the cases of the thousands who frequent some of our hospitals, but the fact that such a man is employed will doubtless help to keep many of the more unworthy from asking help at that particular institution; and thus indirectly he may fulfil his mission. Originally the out-patient room was intended for those who had been dismissed from the hospital, but whom the doctor who had attended them there wished to see occasionally, for fear of relapses or complications. In those days the patients were fewer; the doctor, knowing their case, could diagnose in a few minutes any new development, and recommend a treatment with some confidence. But now the number of patients is immensely increased; the majority of the cases are new; they require more careful examination than the old patient, who was known to the doctor; and, as a matter of fact, he has less time to give them. When, as is often the case, the disease is due to poverty, the patient would be better to go to the poor-law medical officer, who has power to order food and stimulants when he thinks they are needed. In most cases, the patient is able to go to a private practitioner; and it is not fair that hospitals should employ the donations of the wealthy in relieving those who are able to help themselves. It is true that there are many who can keep themselves while in health, who find themselves unable to cope with the emergencies of sickness; but, as sickness is one of those things which every family may well regard as inevitable sooner or later, the principle of insurance should come in. It costs but little to belong to a provident dispensary, and in sickness the small subscription is amply repaid. As Mr. Timothy Holmes points out, the main recommendation of these dispensaries is that "they give a poor man the services of a family doctor, who knows the history and constitution of each member of the family, advises them in all their habits and occupations, and visits them at home when necessary." For this the outpatient room of the hospital and a bottle of medicine given after a two-minute consultation offer no equivalent. Yet there is a place for the out-patient department. If among the poorer patients of any practitioner a case occurs in which, were he richer, the medical attendant would recommend a consultation, he should be able to send him instead to a hospital, where a physician or surgeon, specially skilled in the kind of disease he is suffering from, would examine the case, receive him as an in-patient if that were desirable, or advise the ordinary practitioner as to treatment if that were sufficient. Some such restriction of the out-patient system must come soon, and it is possible that the Prince of Wales's Hospital Fund may be the means of bringing it about. Sir William Broadbent believes that the committee who distribute the fund will form a kind of hospital board, who will make careful inquiry as to the management of the hospitals which they assist; not giving a large donation merely because a large number of patients are treated in any institution, without careful inquiry as to the discrimination shown in admitting patients. As so many of the contributors to this important fund are men of business, we may reasonably expect to see it administered in a way which will guide others in the difficult path of hospital reform.

Hospitals for infectious disease stand on a different platform from those we have been discussing. They are rate-supported for a very good reason, that they form an insurance to the community at large that infectious disease will not be allowed to spread. The cure of the patient who is treated in them is considered; but also the mischief he might do to his fellows if he were not received and treated in them. There should be as much kindness and charity in the management of them as in any general hospital, or even more, for the patient is taken there whether he will or no, and is kept there for reasons with which he is only passively connected. Unfortunately it is in these very hospitals that scandals have most often taken place. Fever hospitals are, from the nature of the disease, less subject to public inspection than others; and perhaps they need it more. In large towns, where there is always a certain amount of infectious disease, the isolation hospitals are generally well managed; but in small country hospitals, where long periods of time intervene between one epidemic and another, the staff are apt to get demoralised by idleness, especially where the nurses are not drawn from a class whose ideals are as high as those who staff the general hospitals. But in this respect there has been a great improvement of late years, and it still continues. The increasing knowledge of and respect for sanitation which is spreading through all ranks of the community is creating a public opinion that tends to keep all hospitals at a high level of efficiency.

---

A young medical woman recently appointed on the staff of an insane asylum in the North expresses some astonishment at the fact that she is entitled to an allowance of 12 bottles of beer each week. Doubtless, the regulation was framed before the possibility of femininity being added to the staff existed. This lady medico, being a strict teetotaler, is rejoicing that her abstention from malted liquors saves the asylum the price annually of 624 bottles of beer.

